# A hybrid educational approach to service learning: impact on student attitudes and readiness in working with medically underserved communities

**DOI:** 10.1080/10872981.2022.2122106

**Published:** 2022-09-18

**Authors:** Arvind Suresh, Nakia M. Wighton, Tanya E. Sorensen, Thomas C. Palladino, Roshini C. Pinto-Powell

**Affiliations:** Department of Medical Education, Geisel School of Medicine at Dartmouth, Hanover, NH, USA

**Keywords:** Social welfare, community health services, medically underserved area, undergraduate medical education, problem-based learning, social determinants of health

## Abstract

Medical students engage with medically underserved communities (MUC) and vulnerable populations but often lack preparation to advocate appropriately for these communities. While preclinical programs with an experiential community component effectively increase knowledge about serving MUC, the pandemic limited clinical opportunities in community settings for learners. We examined the impact of a streamlined, hybrid service learning curriculum on first-year medical student attitudes towards MUC and their readiness and interest in addressing health barriers faced by this population. The redesigned curriculum for the student-led program required participants to attend nine virtual seminars led by faculty and community members with expertise serving MUC. Students partnered with one of three community agencies to organize service projects and gain exposure to the life experiences of MUC using virtual and in-person approaches. Of the fifteen first year medical students who participated in the program, positive attitudes were sustained across all scales using the Medical Student Attitudes Toward the Underserved (MSATU) questionnaire after one year. A majority (≥50%) of students reported a large increase in their knowledge of the health challenges faced by underserved populations after each didactic session. Despite the mostly virtual nature of community partnerships, students reported increased confidence in their ability to direct MUC patients to local resources (p < 0.01). The program also had a positive impact on student interest in working with medically underserved patients in the future, with 71% of participants indicating a significant impact on their interest in working in a medically underserved area. Our redesigned elective curriculum provided participants with foundational knowledge to advocate appropriately for underserved populations and demonstrated the efficacy of virtual approaches for community service and service learning. Our findings suggest hybrid and virtual experiential learning opportunities are a viable and non-inferior curricular approach to teaching health equity and community health.

## Introduction

During the last few decades, it has been recognized that marginalized groups in the USA have significantly poorer health outcomes due to limited access to medical care, health disparities, and multiple comorbidities [1–3]. Medically underserved communities (MUC) are defined as specific populations that have a shortage of primary healthcare services or otherwise face unmet healthcare needs [4]. Healthcare providers often feel ill-equipped to care for these populations, relying on on-the-job training and experience [5]. One way to address these disparities is through instruction in undergraduate medical education regarding social determinants of health (SDoH) [6,7]. SDoH are defined by the World Health Organization as ‘social, physical and economic conditions in society that impact upon health’ [8]. Students frequently engage with MUC but often lack preparation to advocate appropriately for these populations [7,9]. While formalized curricula on social determinants of health enhance preparedness in caring for the medically underserved, the methodology on teaching SDoH varies greatly and is often limited by time and funding that precludes sustainability and scalability [7,10].

One successful approach to teach about SDoH in undergraduate medical education focuses on community engagement and experiential learning. To prepare students to integrate patient-centered care with an understanding of the patients’ community in rural and underserved areas, one curricular intervention required second year medical students to complete a chronic disease management project as part of a 4-week community rotation. Significant differences were noted in student attitudes and knowledge regarding non-financial barriers to health care, health disparities in minority populations, and the need to link medical facilities to other community services [11]. Another model is a longitudinal underserved care pathway for students in clinical years to promote engagement with community organizations and service projects in parallel with preceptorships [12]. Similar gains in student self-reported knowledge, skills, and attitudes were seen for participants in both models.

A major challenge in undergraduate medical education is the decline in medical student attitudes toward the underserved throughout medical education. A systematic review and meta-analysis of fifty-five studies revealed that experiential community-based learning and curricula dedicated to social accountability were the most protective educational interventions against this decline [13]. A recent curricular intervention using required longitudinal service learning at the University of Nevada Las Vegas supports this theory. Students were paired with one of over 50 community service organizations to collaborate on service projects, revealing stable and sustained positive attitudes toward the underserved throughout undergraduate medical education [14]. At the Dartmouth Geisel School of Medicine, we developed a longitudinal community health elective for first-year medical students that combined classroom didactics with experiential service learning. This curricular approach has been previously shown to improve short-term medical student attitudes toward the underserved [15].

Since 2020, the COVID-19 pandemic has disproportionately impacted vulnerable communities, heightening the need for a knowledgeable workforce to address systemic barriers [16]. It has also disrupted medical education, including volunteering and immersion in the local community [17]. While preclinical programs with an experiential community component effectively increase MUC knowledge, the pandemic limited these experiential opportunities for learners. Furthermore, knowledge regarding the impact of COVID-related curricular adaptations is limited [18]. In response to the pandemic, we adapted our previously established elective into a hybrid service learning curriculum utilizing virtual and in-person approaches. Here, we evaluate whether this hybrid approach to service learning is effective in preparing first year medical students to care for MUC and address health disparities. We first compare changes in participant attitudes toward the underserved between the hybrid and in-person curricular approaches. Subsequently, we examine the effectiveness of our hybrid curricular model in improving student knowledge, comfort, confidence, and interest in caring for MUC.

## Materials and methods

### Program description

The original community health curriculum at the Geisel School of Medicine (hereafter labeled ‘in-person’) was initiated in August 2016 as an 8-month elective curriculum for first year medical students. The curriculum combined classroom didactics on SDoH and health disparities with experiential community-based learning in a rural environment [15]. The purpose of the program since its inception has been two-fold: 1) provide early exposure to students on how to address SDoH in real-world settings, and 2) promote understanding of challenges experienced by MUC by partnering students with community organizations that directly serve these populations.

The in-person curriculum consisted of 18 didactic sessions and year-long community partnerships (see Reference 14 for more details). In response to the travel and gathering restrictions during the COVID-19 pandemic, the curriculum was adapted to a streamlined, hybrid format (‘hybrid’) that was offered to first year medical students between September 2020 and May 2021. Students attended a total of nine virtual didactic sessions and were matched with one of three community organizations in lieu of one-to-one mentoring relationships that were not possible during the pandemic. A side-by-side comparison of the components of the in-person and hybrid curricula can be found in Table 1.

**Table 1.** Comparison of in-person and hybrid program formats.

In-person Model	Hybrid Model
18 Classroom didactic learning sessions	9 Virtual didactic learning sessions
Documentary screenings Small group discussions with faculty Journal clubs Community member panels	Small group seminars co-led by community members, senior medical students, and faculty Case-based learning sessions led by community health workers and researchers
Longitudinal Community Partnerships	Longitudinal Community Partnerships
One-to-one mentoring relationships between students and community members from local community organizations Group dinners with community members at organization sites	Students matched with one of three community organizations Virtual and in-person service activities following needs assessment

**Figure 1.**
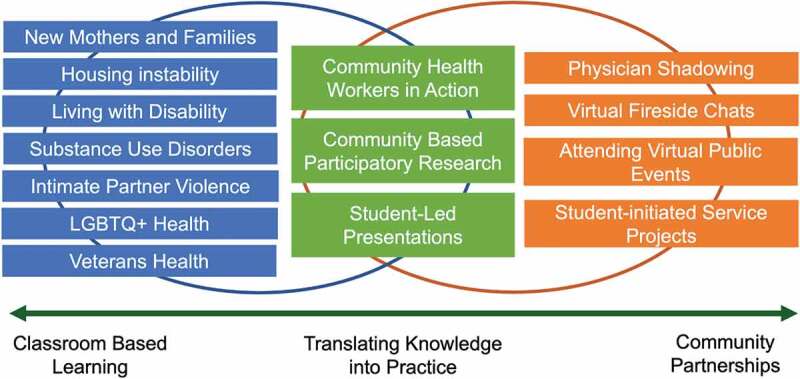
Hybrid service learning elective curriculum framework during the COVID-19 pandemic.

The hybrid program began with seven one-hour virtual learning sessions on various social determinants of health topics that were pre-determined based on community needs (Figure 1). The small group seminars were co-led by community members, senior medical students, and/or faculty with direct experience in serving underserved patient populations. Two novel didactic sessions, ‘Community Health Workers in Action’ and ‘Community Based Participatory Research,’ were added as interactive case-based learning sessions to guide students in applying principles and themes learned from earlier sessions, service learning activities, and community partnerships to real world scenarios. These two sessions were led by local community health workers and researchers with experience conducting community-based participatory research projects in Indigenous communities.

Community partnerships during the pandemic consisted of both virtual and in-person events to connect students with the individuals served by each service organization. Virtual events included fireside chats with community members supported by each organization on conferencing platforms, public education events open to the local community, and physician shadowing via telehealth. In-person community building exercises facilitated by second-year students took place at the beginning and end of the elective to create a space for shared reflection. A full list of sessions and activities in the hybrid curriculum can be found in **Supplementary Appendix 1**. Students initiated a variety of both virtual and in-person service projects. Examples of service projects include online cooking and exercise classes for individuals with disabilities, an outdoor community garden for individuals with disabilities, shifts to restock a local food pantry, and a telehealth program to provide care coordination for homeless individuals during the pandemic (see **Supplementary Appendix 2**).

### Participant selection

Medical students were recruited to participate in the elective through an open application process during the first month of medical school, with enrollment limited only by the capacity of community partner organizations to work with students. All students regardless of background in community health or specialty interest were eligible to participate. As the program was not mandatory for all students, participants in the in-person and hybrid curricula were limited to a self-selected group of students interested in spending additional time outside of the formal curriculum to engage with MUC. Control group participants were recruited via an email to all first-year medical students not participating in the elective curriculum.

### Instruments and outcome variables

#### MSATU

The Medical Student Attitudes Toward the Underserved (MSATU) questionnaire was used to evaluate the impact of both the in-person and hybrid formats of the elective curriculum. The MSATU is a three-section, validated, self-reported measure of medical student attitudes about caring for underserved patient populations [19]. The first section consists of 23 questions that focus on the responsibility of medical students, physicians, government, and charitable organizations to provide healthcare services to underserved populations. Responses from this section comprise the attitudes scale as well as two subscales on societal expectations and professional responsibility. Section 2 consists of 13 questions that survey student opinions regarding whether all patients should have access to medical services. These responses comprise the services scale, which is also divided into subscales on basic and costly services. Section 3 consists of 9 questions that ask about factors that influence access to medical care in the USA. Only questions that were the same between versions of the MSATU across the two cohorts were included for the purposes of analysis. All MSATU survey responses are distributed on a 5-point Likert scale (1 = strongly disagree, 5 = strongly agree). Only the first two sections are used to determine the total MSATU score, calculated as the mean of the first 36 questions. Mean scores for the attitudes and services scales, their component subscales, as well as the total MSATU score were calculated as outcomes for the in-person and hybrid curriculum cohorts.

#### Program evaluation

In addition to completing the MSATU, students in the hybrid program cohort were asked to complete an additional program evaluation survey. At program conclusion, students were asked to evaluate each of nine virtual didactic sessions on the degree to which it increased their knowledge of health challenges and barriers faced by underserved populations (1 = not at all, 5 = extremely). This distribution of scale ratings was used to measure the degree of increase in knowledge regarding medically underserved populations for each didactic session.

Students were also asked at the beginning and end of the program to rate their comfort in talking with individuals from nine MUCs about social determinants and confidence in directing individuals from seven MUCs to local services on a 5-point Likert scale (1 = not at all, 5 = extremely). Comfort discussing social determinants of health with patients was measured using the mean comfort rating score for each MUC, and confidence in directing patients to local community resources was similarly measured using the mean confidence rating score for each MUC. Lastly, students were asked to characterize the impact of the program on future activities related to MUC, as well as to share reflections about their community partnership and the most valuable part of the program in two open-ended questions. Themes and exemplar quotes were subsequently identified using written responses to qualitatively measure the impact of our hybrid service learning elective. The full list of program evaluation questions used for analysis can be found in **Supplementary Appendix 3**.

### Data collection

Data for the in-person program was collected as part of previously published work between August 2016 and April 2017 [15]. As part of this study, first year medical students in the in-person program (n = 13) and a control group (n = 29) were asked to complete the MSATU at the beginning and end of the first year of medical school, during which period the longitudinal elective curriculum took place. Original data from this study was reanalyzed for the purposes of comparison with the hybrid program cohort.

Similarly, all hybrid program participants (n = 15) completed the MSATU at the beginning and end of their first year of medical school. Students in the hybrid cohort also completed an additional program evaluation survey during the two timepoints. Data collection for the hybrid cohort took place during September 2020 and May 2021. All surveys were administered through Qualtrics during a 3-week period and data were collected anonymously. The study was reviewed by the Dartmouth Committee for the Protection of Human Subjects and determined to be exempt from human subjects’ research during both study periods.

### Statistical analysis

As our small sample size limited statistical power, we report statistical tests for key study comparisons and used descriptive statistics and effect sizes to describe other findings. Pearson’s chi square test was used to compare baseline demographic characteristics between the three study groups. We calculated mean scores for each of the scales and subscales of the MSATU, as well as the total MSATU. Linear mixed models were used to examine differences between the two timepoints (pre-program and post-program) for attitudes measured by the MSATU, as well as comfort and confidence in working with MUC measured by items on the hybrid program evaluation questionnaire. We used Cohen’s d_z_ to quantify the magnitude of the change in attitudes, comfort, and confidence [20]. Standard effect size interpretation guidelines contextualized the impact of the in-person and hybrid programs [21]. SAS/STAT software version 9.4 [22] and SPSS version 27.0 [23] were used for all statistical analyses.

## Results

Fifteen students in the hybrid cohort and thirteen students in the in-person cohort completed the pre-program and post-program surveys, yielding a 100% completion rate. Thirty-seven percent of the first year medical student controls who were recruited to take part in the study completed both surveys. Demographic characteristics for each of the study groups are listed in Table 2. The age of study participants was similar across all three study groups. The in-person curriculum cohort comprised a larger percentage of women when compared with the hybrid curriculum cohort (85% vs. 53%) and was also more ethnically diverse (54% Caucasian and 15% African American vs. 73% Caucasian and 0% African American). A similar percentage of students in the in-person and hybrid curriculum cohorts had been involved in projects caring for the medically needy prior to medical school (85% vs. 73%), which was higher than involvement in the control group (52%).

**Table 2.** Demographic information for study participants.^a^

Demographic Characteristics	Hybrid (n = 15)	In-person (n = 13)	Control (n = 29)	p
Gender				
Male	5 (33%)	2 (15%)	11 (38%)	.23
Female	8 (53%)	11 (85%)	18 (62%)	
Other	1 (7%)			
Unanswered	1 (7%)			
Average Age, years	24.4	25.6	24.9	.29
21 to 23	4 (27%)	4 (31%)	13 (45%)	
24 to 26	10 (67%)	7 (54%)	11 (38%)	
27 to 29	1 (7%)	1 (8%)	5 (17%)	
30+	0 (0%)	1 (8%)	0 (0%)	
Race/Ethnicity				
Caucasian	11 (73%)	7 (54%)	20 (69%)	.13
African American	0 (0%)	2 (15%)	0 (0%)	
Other	3 (20%)	3 (23%)	9 (21%)	
Unanswered	1 (7%)	1 (8%)	0 (0%)	
Have been involved in projects providing care to the medically needy	11 (73%)	11 (85%)	15 (52%)	.09

^a^Previously presented in [15] for In-person and Control cohorts.

**Figure 2.**
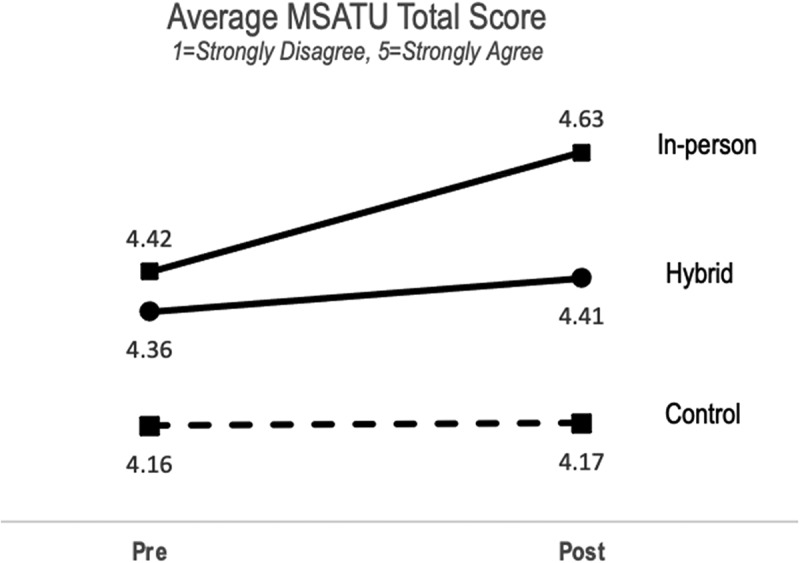
Mean total medical student attitudes toward the underserved (MSATU) score for hybrid and in-person curriculum cohorts.

To evaluate the effect of the in-person and hybrid program formats on participant attitudes toward the underserved, we compared MSATU pre- and post-scores using linear mixed modeling for each program (Figure 2). There were increases in average MSATU total scores post-program in both the in-person (p = .08, d_z_ = 0.53) and hybrid (p = .37, d_z_ = 0.25) programs. There was little change in MSATU scores with the control group (p = 0.78, d_z_ = 0.05).

Comparatively, the average MSATU pre-program scores for the in-person and hybrid programs were similar (4.42 vs. 4.36, d = 0.18), and substantially higher than the control group (d_in-person vs. control_ = 0.62 and d _hybrid vs. control_ = 0.45, respectively). Post-program MSATU average scores were higher for the in-person program than the hybrid program (d = 0.62). Higher total scores, indicative of positive attitudes toward the underserved, were sustained during the first year of medical school for both the hybrid and in-person curriculum cohorts.

Means corresponding to each scale and subscale in the MSATU for the in-person and hybrid program cohorts can be seen in Table 3. Few differences between the pre-program and post-program means reached statistical significance at p < .05 except for the basic services subscale for the in-person program cohort. Despite a lack of statistical significance, both programs demonstrated positive changes in the attitudes scale and subscale scores. Only the in-person program cohort demonstrated improvement in services scale scores, with negligible changes in the hybrid program cohort. The effect sizes show that changes were larger across all MSATU scales and subscales for the in-person program cohort when compared with the hybrid cohort. Among factors influencing access to medical care in Section 3 of the MSATU, there was a significant increase in the number of hybrid program participants reporting that access to care is influenced by urban vs. rural residence ($\underline{x}$ = 4.5 vs. 4.9, p = 0.019).

**Table 3.** Mean pre- and post-scores and Cronbach’s Alpha for MSATU total scale and subscale scores for in-person (n = 13) and Hybrid (n = 15) program formats.^a^

Section	Pre	Post	Effect Size (d_z_)
**Total (α = .91)**			
In-person	4.42	4.63†	0.53
Hybrid	4.36	4.41	0.24
**Attitudes (α = .83)**			
In-person	4.33	4.51†	0.51
Hybrid	4.27	4.37	0.37
*Societal Expectations* (α = .57)			
In-person	4.24	4.51†	0.53
Hybrid	4.20	4.33	0.36
*Professional Responsibility* (α = .83)			
In-person	4.39	4.50	0.40
Hybrid	4.32	4.39	0.23
**Services (α = .95)**			
In-person	4.74	4.95	0.40
Hybrid	4.74	4.67	−0.16
*Basic Services* (α = .98)			
In-person	4.90	5.00*	0.61
Hybrid	4.78	4.81	0.10
*Costly Services* (α = .86)			
In-person	4.58	4.90	0.35
Hybrid	4.71	4.53	−0.29

^a^Cronbach’s Alpha calculated for Pre-program Scores

†p < .10, *p < .05

**Figure 3.**
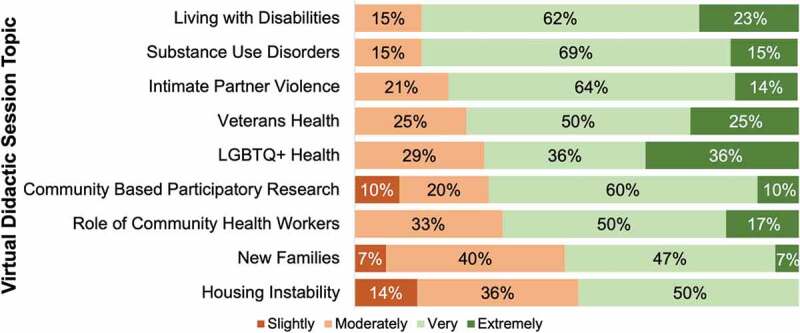
Degree of increase in knowledge of health challenges and barriers faced by medically underserved populations for each virtual didactic session.

To study the overall effectiveness of the hybrid program, we measured the impact of program components on student knowledge of health challenges faced by MUC, comfort discussing social determinants with patients, and confidence directing patients to local community resources. All students in the hybrid curriculum cohort (n = 15) completed a set of program-specific questions in addition to the MSATU. For each virtual didactic session, a majority (>50%) of students reported a large increase in their degree of knowledge of the health challenges and barriers faced by various medically underserved populations (Figure 3).

Students also completed questions regarding their comfort discussing various social determinants and confidence in directing individuals to local resources at both the beginning and conclusion of the program (Table 4). Overall, we found a significant increase in student self-reported confidence in directing patients affected by social determinants to local resources ($\underline{x}$ = 2.31 vs. 3.02, p = 0.007), but no such increase in student comfort discussing these social determinants with patients ($\underline{x}$= 3.26 vs. 3.33, p = 0.72).

**Table 4.** Impact of hybrid elective curriculum on student comfort and confidence in working with medically underserved communities.

	Pre	Post	Effect Size (d_z_)
Comfort discussing social determinants of health with patients^a^ *Average of 9 items*	3.26	3.33	0.10
Homelessness	3.47	3.53	0.07
Disability	2.87	3.13	0.25
Parenting or life with a new child	3.27	3.13	−0.15
Intimate partner or domestic violence	2.47	3.13*	0.62
Substance use and addiction	3.20	3.43	0.20
Sexual orientation	3.60	3.67	0.12
Gender identity	3.53	3.67	0.22
Race and ethnicity	3.47	3.07	−0.39
Cultural background	3.47	3.13	−0.31
Confidence in directing patients affected by social determinants to local community resources^b^ *Average of 7 items*	2.31	3.02**	0.84
Homelessness	2.40	2.87	0.45
Disability	1.93	2.60*	0.66
Parenting or life with a new child	2.80	3.13	0.31
Intimate partner or domestic violence	2.00	3.67**	1.55
Substance use and addiction	2.53	3.27†	0.55
Gender identity	2.12	3.13*	0.75
Race and ethnicity	2.33	2.47	0.10

^a^Rated on a 5-point Likert scale, 1 (Not at all comfortable) to 5 (Extremely comfortable)

^b^Rated on a 5-point Likert scale, 1 (Not at all confident) to 5 (Extremely confident)

†p < 0.10, *p < 0.05, **p < 0.01

**Figure 4.**
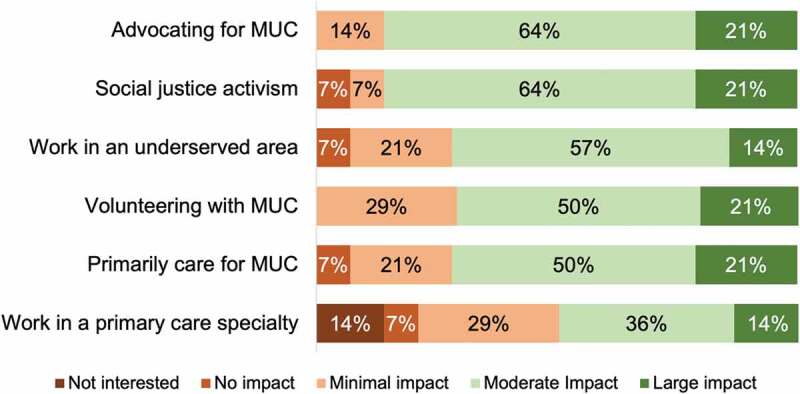
Impact of hybrid service learning curriculum on future interest in working with medically underserved communities.

Most students also indicated that the hybrid program had a moderate or large impact on their interest in advocating for MUC (85%), engaging in social justice activism (85%), and volunteering with underserved populations in the future (71%) (Figure 4). A similar proportion of students also reported that the program significantly impacted their interest in primarily caring for MUC or working in a medically underserved area (71%), regardless of their intended specialty. However, only half of respondents reported a moderate or large impact on their interest in a primary care specialty, and two students shared that they were not interested in a primary care specialty after participating in the program. Despite the constraints of hybrid community partnerships, 67% indicated continued interest in working with their organization throughout medical school. Qualitative student feedback regarding the hybrid curriculum was focused around three themes: understanding the patient perspective, preparing for patient care in future clinical settings, and the ability to connect and learn more about community resources for underserved patient populations. Themes and representative student comments are listed in Table 5.

**Table 5.** Qualitative student comments on impact of hybrid service learning elective curriculum.

Theme	Representative Comments
Understanding patient perspective	*‘I think we are so quick to judge and often slow to remember all the system barriers that prevent people from seeking health-promoting choices.’*
	*‘I have truly learned a lot from the candid conversations that invited speakers have had with us about their observations and experiences [with] medically underserved people … ’*
Preparing for patient care	*‘I am beginning to have a better understanding of the kinds of questions and considerations I should have when caring for an individual with disabilities …’*
	*‘I am excited to continue to build long-term relationships and connect patients with helpful resources in our community that go beyond what we can do in a 20-minute appointment in clinic.’*
Connecting with community resources	*‘[The community partnership] taught me the importance of community support and home visits for new families and encourages me to always know my local resources/have lists of resources available for patients.’*
	*‘The most valuable thing I learned is that there are tons of different community-based programs that are built to help. If someone comes to me with a problem now, I may not have the direct knowledge to fix it but I likely can refer them to people who can.’*

## Discussion

Over a one-year period, our hybrid curricular model was successful in sustaining positive attitudes toward the underserved. First-year medical students reported increased knowledge of the challenges faced by MUCs and confidence in working with MUCs in a clinical setting. The hybrid curriculum also positively impacted student desire to work with underserved populations in the future, demonstrating the efficacy of virtual approaches for community service and service learning in undergraduate medical education.

Our study results suggest that regardless of the format, it is the tangible interaction of medical students with MUC that has the most meaningful impact on student attitudes, knowledge, and readiness. This is supported by prior studies in which only service learning activities that had direct interaction with underserved communities were successful in sustaining medical student attitudes toward the underserved [14]. For example, students in our hybrid cohort increasingly rated rurality as a significant factor impacting access to medical care after hearing stories from community members living in food deserts without reliable transportation. Despite very limited in-person opportunities to connect with community members in the hybrid format, student qualitative feedback highlighted an increased eagerness and preparedness in working with MUC. As transportation and limited time are commonly reported student and community partner barriers to community engagement [24,25], the adoption of hybrid experiential and service learning curricula in the post-pandemic setting has the potential to maximize these learning opportunities for medical students. The use of virtual platforms places less burden on community organizations to provide adequate space and resources for student volunteers, while also expanding the geographic scope of organizations available to partner with medical schools.

The inclusion of content on social determinants of health has become a critical component of undergraduate medical education in recent years, even more so during a pandemic that has deepened pre-existing health disparities [16]. Many studies have suggested that medical training negatively impacts medical student attitudes toward the underserved [13,19,26,27], creating a need for novel approaches to introduce topics of social medicine [28] and health equity to medical students. As previously reported, our medical school’s community health curriculum adopted an experiential learning model in the form of partnerships with local community organizations to equip students with the knowledge and empathy necessary to intervene on behalf of medically underserved communities. Short-term outcomes from this elective curriculum were shown to improve medical student attitudes toward the underserved over a one-year period [15]. In comparison, positive student attitudes toward the underserved persisted or increased in our streamlined, hybrid elective curriculum among all the scales and subscales of the MSATU. Considering data suggesting an expected decline in medical students’ attitudes between the first and second years of medical education [26], outcomes from both the in-person and hybrid cohorts support that experiential learning curricula are successful in maintaining positive attitudes toward the underserved, even when delivered in virtual or hybrid settings.

Despite sustained positive attitudes toward the underserved in the hybrid cohort, post-program MSATU scores remained lower when compared to the in-person cohort. While this might have been expected, possible reasons for this difference include the limited capacity of community organizations to engage with students during the pandemic given increased demand, and the reduced number of didactic sessions in the hybrid cohort (9 vs. 18). Based on student feedback, future iterations of our curriculum will increase the number of community partner agencies and provide more opportunities for student reflection and sharing of experiences.

While student participants reported improved confidence in directing underserved individuals to community resources, there was no corresponding improvement in student comfort discussing SDoH with patients. It is plausible that students entering the curriculum may have already had a high level of comfort in this area based on their prior experiences working with the underserved. Alternatively, students may have also felt that the virtual group discussion setting, especially with the lack of structured one-to-one conversations with community members, was not sufficient to fully prepare them for future patient encounters. Interestingly, our cohort showed a decrease in participant comfort discussing race/ethnicity and cultural background with patients despite showing increased comfort with other SDoH. We hypothesize this may be due to less racial and ethnic diversity in the hybrid cohort, and reflective of increased student awareness of their own knowledge gaps and biases without sufficient experience in addressing these topics with patients. This represents an opportunity for improvement in our curriculum to include additional experiential learning such as simulated patient encounters.

We acknowledge several limitations in our study. The small sample size for both the in-person and hybrid cohorts means we are underpowered and limits our ability to statistically detect modest changes in attitudes, knowledge, or readiness in working with MUC. Additionally, our elective curriculum may have selection bias toward students with an interest in community health and working with underserved populations, limiting the generalizability of our data to all medical students. From its inception, our service learning program was designed to supplement the formal curriculum for students wishing to receive additional training and experience in working with MUC. Our medical school has evolved in recent years to include discussion of healthcare disparities and working with underserved populations as part of a longitudinal curriculum, yet these topics are often incorporated into existing sessions primarily focused on the basic sciences. Further, this curricular approach does not include individual relationships with underserved populations or the organizations that serve them. With medical schools largely moving toward shorter, more condensed preclinical phases, a clear benefit would need to be shown for a curriculum such as ours to be widely adopted. Including all preclinical medical students in an in-person model may also place an increased burden on community partner organizations that may limit sustainability. The viability of a hybrid model that our study demonstrates may alleviate some of this burden and support the future expansion of our program to include all preclinical medical students.

Overall, we are encouraged by the outcomes in this study. When opportunities to learn from community members with lived experience of social determinants of health are included in the curriculum, our findings suggest that hybrid and virtual approaches to service learning are non-inferior to in-person learning. Early exposure to health disparities and social determinants that impact the care of MUC positively impacts student interest in community service and advocacy and empowers students with knowledge of resources they can share with patients. It is also important for student professional development, especially for students who may be considering a career in primary care and are beginning to explore medical specialties. Future studies are necessary to evaluate the long-term impact of such curricular interventions, and the sustainability of hybrid and virtual experiential techniques in undergraduate medical education.

## Conclusions

Experiential and service learning opportunities during medical school have been shown to improve medical student attitudes toward underserved patient populations. Our study demonstrates that direct interaction with individuals who have lived experience, even in a virtual setting, impacts medical students’ attitudes toward the underserved and prepares students to address the needs of medically underserved communities in their future practice. These findings have implications in expanding the scope of experiential service learning curricula and informing the creation of hybrid curricula on topics of health equity that promote student compassion, interest in service toward medically underserved communities, and career exploration in primary care.

## Supplementary Material

Supplemental Material
