# Supplementary material for: A hybrid educational approach to service learning: impact on student attitudes and readiness in working with medically underserved communities
Source: Med Educ Online. 2022 Sep 18;27(1):2122106. doi: 10.1080/10872981.2022.2122106 (PMC9518252; doi:10.1080/10872981.2022.2122106)
Supplement: Supplemental Material [file ZMEO_A_2122106_SM5657.docx]

**Supplementary Appendix 1.** Hybrid service learning program activities in chronologic order

| Date | Session Title | Activity Type |
| --- | --- | --- |
| September 6 | Community Building Exercises #1 | In-person gathering |
| September 8 | Introduction to the Program | Virtual orientation |
| September 11 | Community Building Exercises #2 | In-person gathering |
| September 12 | Community Building Exercises #3 | In-person gathering |
| September 15 | Challenges Faced by New Families in the Upper Valley with Denise Gariepy | Virtual small group seminar |
| October 6 | Housing Instability in the Upper Valley & COVID-19 with Dr. John Sanders | Virtual small group seminar |
| October 20 | Living with Disabilities in the Upper Valley with Melissa Norton | Virtual small group seminar |
| October 28 | Introduction to the Upper Valley Haven with Faith DuBois and community members | Virtual community partnership activity |
| November 17 | Substance Use and Addiction with Aya Bashi and Kennedy Jensen | Virtual small group seminar |
| November 20 | Spark! Community Center Thanksgiving celebration | Virtual community partnership activity |
| December 4 | Good Beginnings Introductory Training with Denise Gariepy | Virtual community partnership activity |
| December 10 | Virtual Fireside Chat with Upper Valley Haven community members | Virtual community partnership activity |
| December 15 | Intimate Partner and Domestic Violence in the Upper Valley with Dr. Patricia Glowa and Kate Rohdenburg | Virtual small group seminar |
| January 12 | LGBTQ+ Health and Transgender Care with Dr. Benjamin Boh and Bob Linscott | Virtual small group seminar |
| January 15 | Spark! Community Center Bingo Night | Virtual community partnership activity |
| January 17 | Upper Valley Haven Food Pantry Volunteering #1 | In-person service activity |
| January 18 | Upper Valley Haven Food Pantry Volunteering #2 | In-person service activity |
| January 29 | “Mentors of Moms” Volunteer Training Session with Denise Gariepy | Virtual community partnership activity |
| March 9 | Veterans’ Health with Dr. Emily Cohen, Dr. Joel Bradley, and Dr. Don Kollisch | Virtual small group seminar |
| May 13 | Student Participant Presentations #1 | Virtual student presentations |
| May 17 | Community Health Workers and Real-World Problem Solving with Natalie Romano and Lindsey Boisvert | Virtual case-based learning session |
| May 21 | Student Participant Presentations #2 | Virtual student presentations |
| May 26 | Community Based Participatory Research with Dr. Gail Dana-Sacco | Virtual case-based learning session |
| June 8 | Student Participant Presentations #3 & end of program reflections | In-person gathering |

**Supplementary Appendix 2.** Longitudinal service activities by student participants in hybrid program

| Community Organization | Service Activities |
| --- | --- |
| Upper Valley Haven | In-person restocking of food pantry  Virtual care coordination for community members |
| Spark! Community Center | Virtual bi-weekly movement classes  Virtual bi-weekly cooking classes  Started in-person community garden with community members |
| Good Beginnings of the Upper Valley | Accompany volunteers on virtual home visits |

**Supplementary Appendix 3.** Program evaluation survey questions for hybrid curriculum cohort. Only questions analyzed in study results are included. For a copy of questions asked in the MSATU, please contact survey creator Dr. Sonia Crandall.

***Pre-Program Survey Questions***

1. Indicate your level of **comfort** talking with individuals about how the following impacts their health:

- Living situations
  - Not at all comfortable
  - Slightly comfortable
  - Moderately comfortable
  - Very comfortable
  - Extremely comfortable
- Disability
  - Not at all comfortable
  - Slightly comfortable
  - Moderately comfortable
  - Very comfortable
  - Extremely comfortable
- Parenting of life with a new child
  - Not at all comfortable
  - Slightly comfortable
  - Moderately comfortable
  - Very comfortable
  - Extremely comfortable
- Intimate partner of domestic violence
  - Not at all comfortable
  - Slightly comfortable
  - Moderately comfortable
  - Very comfortable
  - Extremely comfortable
- Substance use and addiction
  - Not at all comfortable
  - Slightly comfortable
  - Moderately comfortable
  - Very comfortable
  - Extremely comfortable
- Sexual orientation
  - Not at all comfortable
  - Slightly comfortable
  - Moderately comfortable
  - Very comfortable
  - Extremely comfortable
- Gender identity
  - Not at all comfortable
  - Slightly comfortable
  - Moderately comfortable
  - Very comfortable
  - Extremely comfortable
- Race and ethnicity
  - Not at all comfortable
  - Slightly comfortable
  - Moderately comfortable
  - Very comfortable
  - Extremely comfortable
- Cultural background
  - Not at all comfortable
  - Slightly comfortable
  - Moderately comfortable
  - Very comfortable
  - Extremely comfortable

2. Indicate how **confident** you are in your ability to direct someone to local services and resources if they shared the following with you:

- “I don’t know where to sleep tonight”
  - Not at all confident
  - Slightly confident
  - Moderately confident
  - Very confident
  - Extremely confident
- “I’m struggling to cope with everyday life due to my disability”
  - Not at all confident
  - Slightly confident
  - Moderately confident
  - Very confident
  - Extremely confident
- “I’m struggling to take care of my newborn”
  - Not at all confident
  - Slightly confident
  - Moderately confident
  - Very confident
  - Extremely confident
- “I was physically assaulted by my partner and I’m afraid to go back home”
  - Not at all confident
  - Slightly confident
  - Moderately confident
  - Very confident
  - Extremely confident
- “I don’t want to be addicted to opioids anymore”
  - Not at all confident
  - Slightly confident
  - Moderately confident
  - Very confident
  - Extremely confident
- “I no longer identify with the sex I was assigned at birth”
  - Not at all confident
  - Slightly confident
  - Moderately confident
  - Very confident
  - Extremely confident
- “I feel that I was treated poorly because of my race or cultural background”
  - Not at all confident
  - Slightly confident
  - Moderately confident
  - Very confident
  - Extremely confident

***Post-Program Survey Questions***

1. Below is a list of the didactic sessions that took place during the 2020-21 academic year as part of the Community Health Scholars program. Rate how much each session increased your knowledge of the health challenges and barriers often faced by medically underserved and vulnerable populations.

- 9/15 - Challenges Faced by New Families in the Upper Valley
  - Not at all
  - Slightly
  - Moderately
  - Very
  - Extremely
  - N/A - Did not attend
- 10/6 - Housing Instability in the Upper Valley & COVID-19
  - Not at all
  - Slightly
  - Moderately
  - Very
  - Extremely
  - N/A - Did not attend
- 10/20 - Living with Disabilities in the Upper Valley
  - Not at all
  - Slightly
  - Moderately
  - Very
  - Extremely
  - N/A - Did not attend
- 11/17 - Substance Use and Addiction
  - Not at all
  - Slightly
  - Moderately
  - Very
  - Extremely
  - N/A - Did not attend
- 12/15 - Intimate Partner and Domestic Violence in the Upper Valley
  - Not at all
  - Slightly
  - Moderately
  - Very
  - Extremely
  - N/A - Did not attend
- 1/12 - LGBTQ+ Health and Transgender Care
  - Not at all
  - Slightly
  - Moderately
  - Very
  - Extremely
  - N/A - Did not attend
- 3/9 - Veterans Health
  - Not at all
  - Slightly
  - Moderately
  - Very
  - Extremely
  - N/A - Did not attend
- 5/17 - Community Health Workers and Real World Problem Solving
  - Not at all
  - Slightly
  - Moderately
  - Very
  - Extremely
  - N/A - Did not attend
- 5/26 - Community Based Participatory Research
  - Not at all
  - Slightly
  - Moderately
  - Very
  - Extremely
  - N/A - Did not attend

2. Did you engage in any service work or projects with your community partner organization this year?

- Yes
- No

3. Describe in 1-2 sentences how your experience with your community partner organization this year will inform your future practice as a physician.

4. ​​How likely are you to continue working with your community partner organization during the remainder of your time in medical school?

- Very unlikely
- Unlikely
- Unsure
- Likely
- Very likely

5. Now that you have completed the first year program, indicate your level of **comfort** talking with individuals about how the following impacts their health:

- Living situations
  - Not at all comfortable
  - Slightly comfortable
  - Moderately comfortable
  - Very comfortable
  - Extremely comfortable
- Disability
  - Not at all comfortable
  - Slightly comfortable
  - Moderately comfortable
  - Very comfortable
  - Extremely comfortable
- Parenting of life with a new child
  - Not at all comfortable
  - Slightly comfortable
  - Moderately comfortable
  - Very comfortable
  - Extremely comfortable
- Intimate partner of domestic violence
  - Not at all comfortable
  - Slightly comfortable
  - Moderately comfortable
  - Very comfortable
  - Extremely comfortable
- Substance use and addiction
  - Not at all comfortable
  - Slightly comfortable
  - Moderately comfortable
  - Very comfortable
  - Extremely comfortable
- Sexual orientation
  - Not at all comfortable
  - Slightly comfortable
  - Moderately comfortable
  - Very comfortable
  - Extremely comfortable
- Gender identity
  - Not at all comfortable
  - Slightly comfortable
  - Moderately comfortable
  - Very comfortable
  - Extremely comfortable
- Race and ethnicity
  - Not at all comfortable
  - Slightly comfortable
  - Moderately comfortable
  - Very comfortable
  - Extremely comfortable
- Cultural background
  - Not at all comfortable
  - Slightly comfortable
  - Moderately comfortable
  - Very comfortable
  - Extremely comfortable

6. Now that you have completed the first year program, indicate how **confident** you are in your ability to direct someone to local services and resources if they shared the following with you:

- “I don’t know where to sleep tonight”
  - Not at all confident
  - Slightly confident
  - Moderately confident
  - Very confident
  - Extremely confident
- “I’m struggling to cope with everyday life due to my disability”
  - Not at all confident
  - Slightly confident
  - Moderately confident
  - Very confident
  - Extremely confident
- “I’m struggling to take care of my newborn”
  - Not at all confident
  - Slightly confident
  - Moderately confident
  - Very confident
  - Extremely confident
- “I was physically assaulted by my partner and I’m afraid to go back home”
  - Not at all confident
  - Slightly confident
  - Moderately confident
  - Very confident
  - Extremely confident
- “I don’t want to be addicted to opioids anymore”
  - Not at all confident
  - Slightly confident
  - Moderately confident
  - Very confident
  - Extremely confident
- “I no longer identify with the sex I was assigned at birth”
  - Not at all confident
  - Slightly confident
  - Moderately confident
  - Very confident
  - Extremely confident
- “I feel that I was treated poorly because of my race or cultural background”
  - Not at all confident
  - Slightly confident
  - Moderately confident
  - Very confident
  - Extremely confident

7. Please characterize the impact that the CHS program had on your **interest** in engaging in the following activities in the future:

- Volunteering with medically underserved populations
  - I realized I am not interested
  - No impact
  - Minimal impact
  - Moderate impact
  - Large impact
- Advocating to improve healthcare for medically underserved populations
  - I realized I am not interested
  - No impact
  - Minimal impact
  - Moderate impact
  - Large impact
- Activism focused on social justice issues
  - I realized I am not interested
  - No impact
  - Minimal impact
  - Moderate impact
  - Large impact
- Work primarily in a medically underserved area
  - I realized I am not interested
  - No impact
  - Minimal impact
  - Moderate impact
  - Large impact
- Primarily care for medically underserved populations
  - I realized I am not interested
  - No impact
  - Minimal impact
  - Moderate impact
  - Large impact
- Work in a primary care specialty
  - I realized I am not interested
  - No impact
  - Minimal impact
  - Moderate impact
  - Large impact

8. What is the most valuable thing you learned through the Community Health Scholars program this year?
